# Femoro-femoral cardiopulmonary bypass for the resection of an anterior mediastinal mass

**DOI:** 10.4103/0019-5049.72649

**Published:** 2010

**Authors:** Chaitali SenDasgupta, Gautam Sengupta, Kakali Ghosh, Asit Munshi, Anupam Goswami

**Affiliations:** Department of Anaesthesiology, Institute of Post Graduate Medical Education and Research, Kolkata, India; 1 Department of Cardiothoracic & Vascular Surgery, Institute of Post Graduate Medical Education and Research, Kolkata, India

**Keywords:** Anaesthesia, anterior mediastinal mass, femoro-femoral bypass

## Abstract

The perioperative management of patients with mediastinal mass is challenging. Complete airway obstruction and cardiovascular collapse may occur during the induction of general anaesthesia, tracheal intubation, and positive pressure ventilation. The intubation of trachea may be difficult or even impossible due to the compressed, tortuous trachea. Positive pressure ventilation may increase pre-existing superior vena cava (SVC) obstruction, reducing venous return from the SVC causing cardiovascular collapse and acute cerebral oedema. We are describing here the successful management of a patient with a large anterior mediastinal mass by anaesthetizing the patient through a femoro-femoral cardiopulmonary bypass (fem-fem CPB).

## INTRODUCTION

Due to high incidence of respiratory and cardiovascular complications, anaesthetic management of mediastinal mass is very difficult. Here we describe successful management of an anterior mediastinal mass with help of cardiopulmonary bypass.

## CASE REPORT

**Figure 1:**
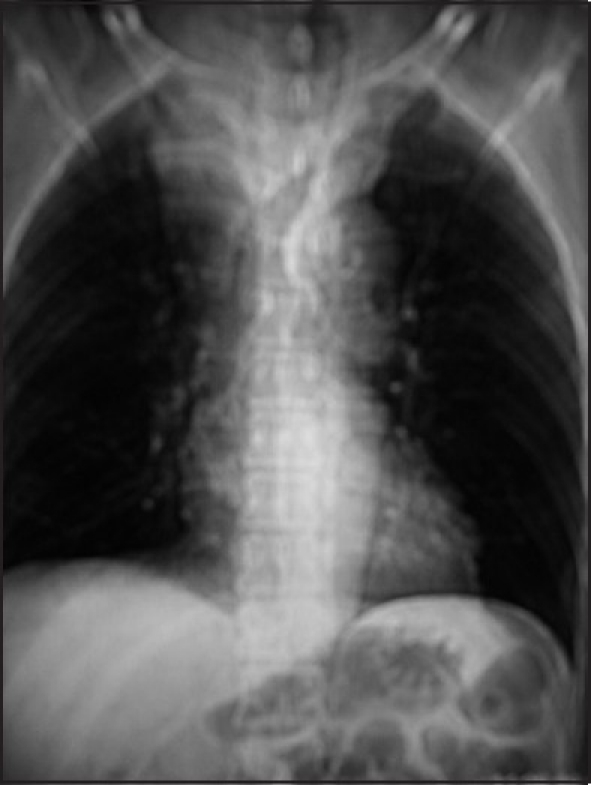
Chest X-ray

**Figure 2:**
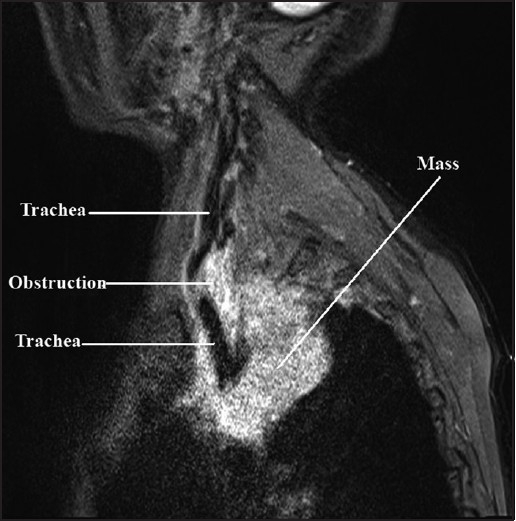
Sagittal view of MRI showing the trachea, obstruction and the mass

**Figure 3:**
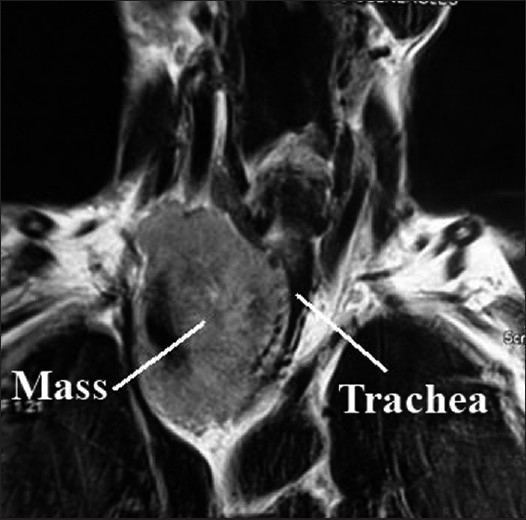
Cross-sectional view of MRI showing the trachea and tortuosity of the mass

A 65 year old male patient was admitted with severe dyspnoea, stridor and cyanosis. In spite of treatment with intravenous antibiotics, corticosteroids, bronchodilator, chest physiotherapy and 100% oxygen inhalation, his SpO_2_, PaO_2_ and PaCO_2_ were 88-91%, 78 torr and 48 torr, respectively. His chest X-ray revealed a large mass on the right side of the chest, shifting and compressing the trachea with widening of the upper mediastinal shadow [Figure 1] The trachea was deviated to left with compression of the right side. His chest MRI [Figures 2 and 3] revealed a retro-sternal, anterior mediastinal solid mass compressing the trachea with critical narrowing of tracheal lumen. Immediate surgical excision of the anterior mediastinal mass was necessary.

It was decided to establish a femoro-femoral cardiopulmonary bypass (CPB) under local anaesthesia, to induce general anaesthesia via CPB for excision of the mass, as endotracheal intubation on induction seemed extremely hazardous, if not impossible even over a fibre-optic bronchoscope.[1]

In the OT, intravenous and arterial cannulae were inserted in the left hand and the central venous cannula was inserted in the left subclavian vein under local anaesthesia. Due to oedema in both lower limbs, IV line could not be inserted in feet. The groins were left for femoral cannulation. The patient was allowed to inhale 100% oxygen continuously. The femoral artery and vein were cannulated in right groin under local anaesthesia. Injection of rocuronium 1 mg/kg, fentanyl 2*µ*g/kg and thiopentone 2 mg/kg were added to the priming fluid in the CPB reservoir. Normothermic CPB was established with a 2.4 L/min flow initially via the femoral route after full heparinization. Just after the onset of CPB, propofol 100 mg was given intravenously. Anaesthesia was maintained with oxygen, air, midazolam, fentanyl and propofol infusion via CPB. Assisted ventilation was possible via an LMA which was introduced after the onset of CPB.

Median sternotomy was done. A firm mass was found in the upper anterior mediastinum and the lower part of the neck engulfing the lower trachea, brachiocephalic trunk and left innominate vein. The mass was subtotally removed to relieve these structures and the trachea was freed from the surrounding mass. After relieving tracheal compression, the LMA was replaced with an endotracheal tube of 8 mm internal diameter. Ventilation was continued via the endotracheal tube thereafter with oxygen and sevoflurane. The patient was weaned off CPB after the completion of surgery.

Monitoring included electrocardiography, pulse oximetry, capnography, thermometry, arterial blood gas, serum electrolytes, activated clotting time, spirometry, respiratory gas analysis, continuous invasive arterial blood pressure and central venous pressure recording.

He was shifted to the intensive care unit and electively ventilated till next morning, was on T-piece for another 24 h and was extubated thereafter. He recovered uneventfully and was discharged from the intensive care unit after 4 days.

## DISCUSSION

In this patient, MRI showed the level of tracheal constriction extending from T1 to T4 vertebrae. The minimum tracheal diameter was 7 mm. The tracheal diameter was 18 mm above the level of constriction and below constriction it was bifurcated into bronchi. The tracheal course was tortuous throughout the constriction.

Tumour compressing airways or great vessels may create a critical respiratory and/or haemodynamic situation. Complete airway obstruction and cardiovascular collapse may occur during induction of general anaesthesia[2], tracheal intubation[3] and positive pressure ventilation.[4] Standard anaesthetic management options include an induction of anaesthesia on an adjustable surgical table, use of short acting anaesthetics, avoidance of muscle relaxants, maintenance of spontaneous respiration during intubation and maintenance and awake intubation by a fibreoptic bronchoscope. The idea of primary endotracheal intubation by a fibreoptic bronchoscope was abandoned here due to extreme tortuosity of the trachea to prevent airway injury and further hypoxia.

During anaesthesia for the endoscopic palliative management of the large anterior mediastinal mass, awake intubation was reported and spontaneous ventilation was maintained with Heliox.[5] Heliox reduces resistance to the airflow through a compressed airway, and maintains oxygenation.[6] Heliox is not available in our centre. Emergency CPB was established in a patient with mediastinal mass when attempts for awake fibreoptic intubation failed.[7] In case of severe clinical symptoms and large mediastinal tumours, cannulation of femoral vessels preoperatively under local anaesthesia and availability of CPB is absolutely essential.[8] A temporary extracorporeal jugulo-saphenous bypass was reported for peri-operative management of a patient with superior vena cava (SVC) obstruction.[9]

Loss of control of the airway was reported by inhalational and intravenous inductions for patients with mediastinal masses. Induction of anaesthesia and muscle relaxation may reduce chest wall tone and may exacerbate airway compression.[10]

So, it was decided to initiate CPB via the femoral route here. The induction of anaesthesia was done with intravenous propofol just before the onset of CPB to prevent awareness. Injections of thiopentone and vecuronium were added to the reservoir after initiating CPB to prevent deoxygenation and awareness at any point of time. LMA was not used before CPB to prevent the loss of airway control with extremely difficult ventilation by general anaesthesia. It was inserted to have an airway access, and to oxygenate blood flowing through the pulmonary circulation, which was a possibility as heart was never arrested and was allowed to beat during CPB. A flexometallic ETT was not used under spontaneous ventilation to prevent airway injury. The ETT could be inserted only after the trachea was released from the mediastinal mass. The patient was ventilated electively till next morning and extubated on the following day. He did not develop tracheomalacia but extubation was delayed to settle down oedema surrounding the trachea following surgery. He was comfortable after extubation, maintained 100% SpO_2_ in room air, and had no memory of surgery.

A CT scan is performed in these cases to identify location, relation to adjacent structures, extent of tracheal or vascular compression and calcification. CT and MRI in a supine position, in pre-anaesthetic evaluation, detect position related compression syndromes. General anaesthesia is safe when CT measured minimum tracheobronchial diameter is >50% of normal in asymptomatic adults; unsafe when <50% of normal regardless of symptoms in children; uncertain in mild/moderate symptomatic children with >50% of normal and mild/moderate symptomatic adult with <50% of normal.[11] MRI is superior to the CT scan as it distinguishes soft tissues from vascular structures, and identifies vascular compression and tissue invasion.[1] Angiography and echocardiography are done if obstruction to the pulmonary artery and/or SVC is suspected. Echocardiography diagnoses pericardial effusion. The peak expiratory flow rate (PEFR) reflects the central airway diameter. A PEFR less than 50% of the predicted in a supine position signifies anaesthetic complication. Maximum inspiratory and expiratory flow volume loops are performed in the supine and standing position of a patient to see fixed and variable airway obstruction. In patients with variable intrathoracic lesions, the inspiratory flow is well preserved and the expiratory flow is diminished, with characteristic flattening of the expiratory loop.

CT guided needle biopsy is done with local anaesthesia in adults. It is difficult in children. In the case of tumours related to vascular structures, biopsy is done under GA by mediastinoscopy, thoracoscopy and mediastinotomy.

If the trachea cannot be intubated beyond the lesion, microlaryngeal endotracheal tube, distal jet ventilation, rigid bronchoscopy along with Venturi injector, intubation of the proximal trachea and temporary stenting or intubation in prone, semi-erect or lateral position may help. In vascular compression of the right heart or pulmonary artery, inotropes and vasoconstrictors with intravascular volume loading are useful. Bleomycin is used sometimes to reduce the size of tumour preoperatively, but may cause pulmonary toxicity. So, pulmonary function test must be done before giving bleomycin. In patients treated with bleomycin, oxygen concentration must be kept low during surgery to prevent pulmonary toxicity.

## CONCLUSION

Preoperative control of the airway seemed to be impossible here, so CPB was used. Femoro femoral CPB was initiated electively because during emergency it is not only difficult to institute but also increases mortality. After the institution of bypass, the LMA was inserted to have a control over the airway and after the removal of the mass, the patient was intubated. He was kept intubated electively for 2 days to settle down oedema of the surrounding soft tissue.

So, when the preoperative control of the airway is apparently impossible, femfem bypass under local anaesthesia may be instituted before induction, to overcome airway obstruction, to get control of ventilation as well as oxygenation without doing any airway injury and without having any incidence of hypoxia.
